# Supplementary material for: CPT1A loss promotes lung metastasis in immune-competent mice via a mechanism of mtDNA release and chronic activation of STING pathway
Source: bioRxiv. 2026 May 5:2026.05.01.722261. Preprint. [Version 1] doi: 10.64898/2026.05.01.722261 (PMC13174484; doi:10.64898/2026.05.01.722261)
Supplement: Supplement 1 [file media-1.pdf]

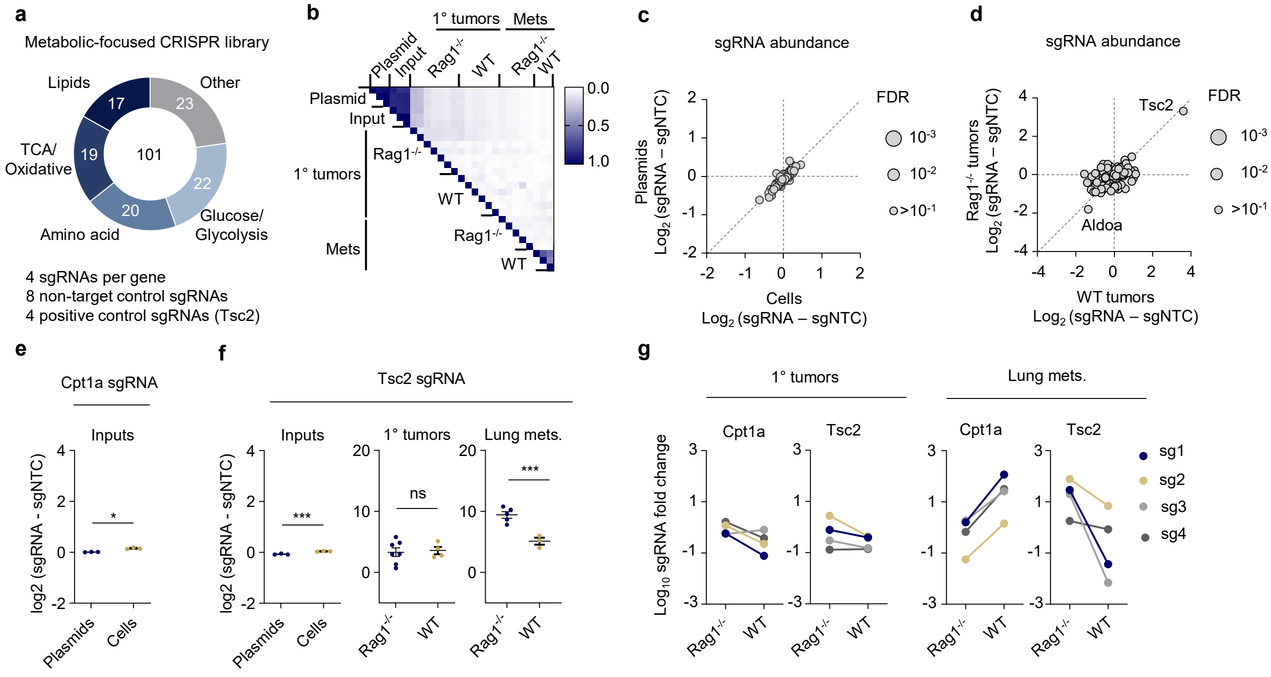

Extended Data.1 | Metabolic-focused *in vivo* CRISPR KO screen identifies CPT1A as an immune-dependent suppressor of metastasis. a, Distribution of genes in the metabolic-focused CRISPR KO library. **b**, Pearson correlation coefficient of the normalized sgRNA read counts across plasmid, input cells when performed orthotopic transplantation (day3 after antibiotic selection), primary tumors (1° tumors, 3 weeks after transplantation) and whole lung with outgrowth metastatic tumors (Lung mets., 4-9 weeks after tumor resection) from both $Rag1^{-/-}$ and BALB/c WT mice. $n \geq 3$ mice per group. **c-d**, The differential abundance of sgRNA corresponding genes between plasmid library and input cells (**c**) or in primary tumors between $Rag1^{-/-}$ and BALB/c WT mice (**d**). Target gene sgRNAs were normalized to non-targeted control sgRNA (sgNTC). **e-f**, Normalized log2-transformed read counts of *Cpt1a* in input cells (**e**) or the tumor growth suppressor *Tsc2* in input cells, primary tumors, or lung metastases (**f**). $n \geq 3$ mice per group. Welch's t-tests were performed. **g**, Log_{10} transformed read counts of individual Cpt1a or Tsc2 sgRNAs in primary tumors and lung metastases from $Rag1^{-/-}$ (n=7) or BALB/c (n=4) mice. *p<0.05, ***p<0.005. ns, not significant.

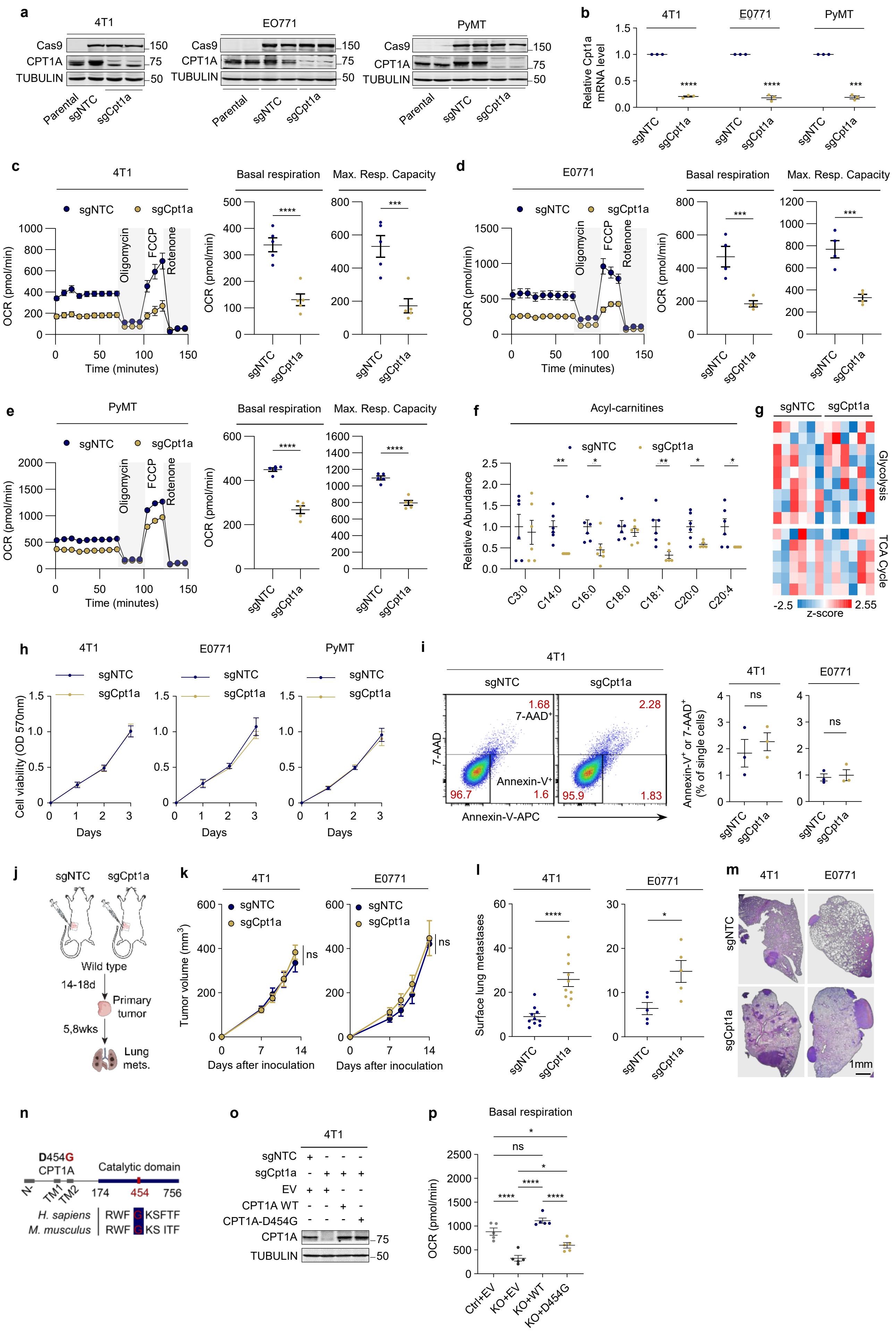
a
4T1
E0771
PyMT
Cas9
CPT1A
TUBULIN
150
75
50
Parental
sgNTC
sgCpt1a
b
Relative Cpt1a mRNA level
****
***
c
4T1
sgNTC
sgCpt1a
OCR (pmol/min)
Time (minutes)
Oligomycin
FCCP
Rotenone
Basal respiration
Max. Resp. Capacity
d
E0771
e
PyMT
f
Acyl-carnitines
Relative Abundance
C3:0
C14:0
C16:0
C18:0
C18:1
C20:0
C20:4
g
Glycolysis
TCA Cycle
-2.5
2.55
z-score
h
Cell viability (OD 570nm)
Days
i
7-AAD
7-AAD+
Annexin-V+
Annexin-V-APC
1.68
96.7
1.6
2.28
95.9
1.83
Annexin-V+ or 7-AAD+ (% of single cells)
ns
j
Wild type
14-18d
Primary tumor
5,8wks
Lung mets.
k
Tumor volume (mm3)
Days after inoculation
ns
l
Surface lung metastases
m
1mm
n
D454G
CPT1A
Catalytic domain
N-
TM1
TM2
174
454
756
H. sapiens
M. musculus
RWF G KSFTF
RWF G KS ITF
o
EV
CPT1A WT
CPT1A-D454G
p
Ctrl+EV
KO+EV
KO+WT
KO+D454G

Extended Data.2 | CPT1A deficiency impairs cellular respiration and increases metastatic tumor volume in the lung. a, Immunoblots of CPT1A and Cas9 in parental, sgNTC control and *Cpt1a*-KO (sgCpt1a) in 4T1, EO771 and PyMT (BL6). **b**, Relative *Cpt1a* mRNA expression (fold change) determined by qPCR in control (sgNTC) and CPT1A-deficient (sgCpt1a) 4T1, EO771 and PyMT cells. Unpaired Student's t-tests were performed. **c-e**, Oxygen consumption rate (pmol/min) of control (sgNTC) or CPT1A-deficient (sgCpt1a) 4T1 (**c**), EO771 (**d**), or PyMT (**e**) cells was determined (per $5x10^4$ cells) (n=4-5) using the Seahorse long-chain fatty acid oxidative stress assay kit. Basal respiration and maximum respiration capacity OCR data are shown. Unpaired Student's t-test was performed. **f**, Relative abundance of acyl-carnitines in control (sgNTC) or *Cpt1a*-KO (sgCpt1a) 4T1 cells. Welch's t-tests were performed. **g**,Heat map showing relative abundance of metabolites associated with glycolysis or TCA cycle in control (sgNTC) or *Cpt1a*-KO (sgCpt1a) 4T1 cells. Welch's t-tests indicated no differences in metabolite abundance between groups. **h**, Control (sgNTC) or Cpt1a-deficient (sgCpt1a) 4T1, EO771 and PyMT tumor cell viability at indicated time points, as measured by MTT cell viability assay (n=5 per group). Two-way ANOVA; p=0.803 (4T1), p=0.124 (EO771), p=0.446 (PyMT). **i**, Apoptosis of control (sgNTC) or Cpt1a-deficient (sgCpt1a) 4T1 or EO771 cells was assessed by Annexin V and 7-AAD staining (n=3). Representative dot plots of 4T1 cells are shown, and unpaired Student's t-tests were performed. **j**, Experimental timeline for *in vivo* lung metastasis evaluation of control (sgNTC) and Cpt1a-*KO* (sgCpt1a) 4T1 or EO771 tumor cells. Primary tumors were resected on day 14-18 post-implantation, and whole lungs were collected 4 weeks and 8 weeks after 4T1 or EO771 tumor resection, respectively. **k**, Primary tumor growth from mice described in (**j**) (n≥5 mice per group). Two-way ANOVA; p=0.369 (4T1), p=0.727 (EO771). **l-m**, Lung metastasis of mice described in (**j**). Number of lung surface metastases in BALB/c-Cas9 mice or BL6-Cas9 (**l**) were determined. Representative H&E images are shown in (**m**). Welch's t-tests were performed. **k**, The D454 site is conserved in human and mouse CPT1A, located within the catalytic carnitine O-palmitoyltransferase domain. The D454G mutant in humans leads to low CPT1A activity. **o**, Immunoblot of CPT1A in Ctrl (sgNTC) (lane 1) or *Cpt1a*-KO (sgCpt1a) cells transduced with empty vector (EV) (lane 2), WT CPT1A (lane 3), or CPT1A D454G (lane 4). **p**, Basal respiration OCR was determined in sgNTC control (Ctrl), *Cpt1a*-KO (KO) 4T1 cells or KO cells re-expressing WT or D454G CPT1A, per $5x10^4$ cells from Figure 1g. One-way ANOVA ($p=8.19x10^{-7}$) with Tukey's post hoc. *p<0.05, **p<0.01, ***p<0.005, ****p<0.001. ns, not significant.

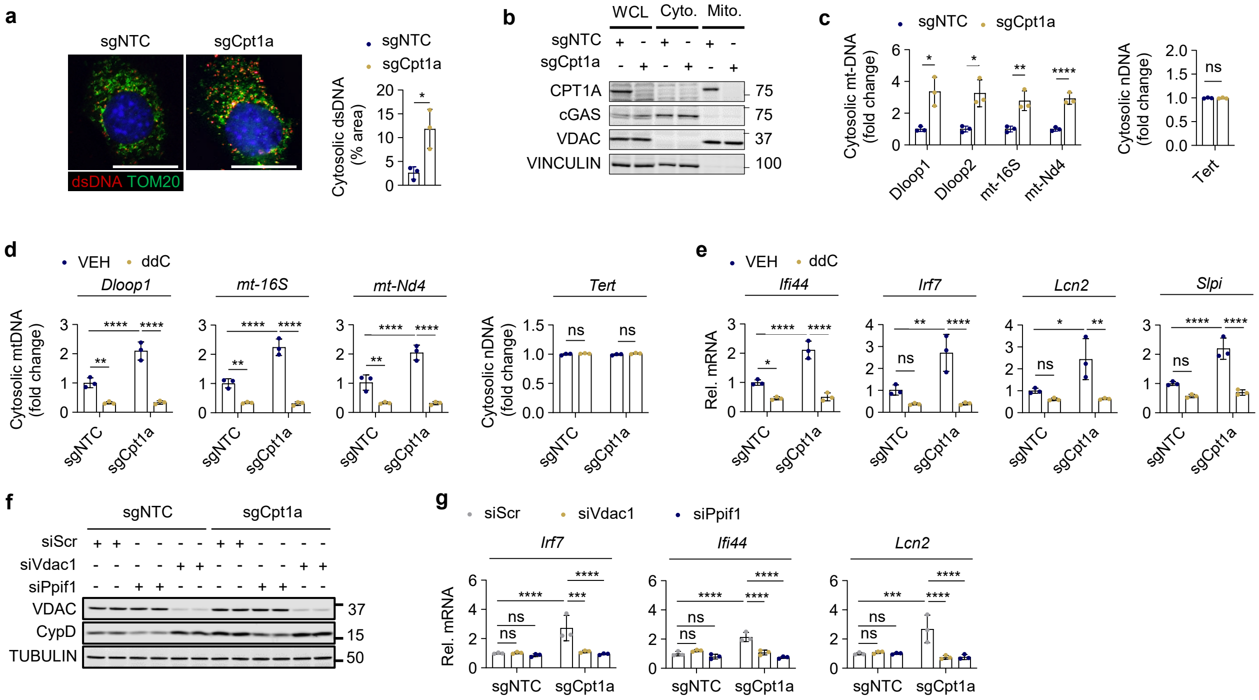

Extended Data.3 | The mPTP pore is required for CPT1A loss driven inflammation. **a**, Immunofluorescence staining of dsDNA (red) and Tom20 (green) in control (sgNTC) and *Cpt1a*-KO (sgCpt1a) PyMT cells. Scale bar 20μM. Cytosolic dsDNA coverage was determined as a percent (%) of total area. Unpaired Student's t-test was performed. **b-c**, Fractionation of PyMT control (Ctrl sg or sgNTC) or *Cpt1a*-KO (Cpt1a sg or sgCpt1a) cells was performed to collect the cytosolic fraction. **b**, Immunoblot to demonstration fractionation. Markers to denote mitochondrial (Mito., CPT1A and VDAC) and cytosolic (Cyto., cGAS and VINCULIN) fractions are shown. Whole cell lysates (WCL) serve as an input control. **c**, Relative levels of mitochondrial DNA (mtDNA) regions (Dloop1, Dloop2, mt-16S, mt-Nd4) (left) or nuclear DNA (nDNA, Tert) (right) in the cytosolic fraction was determined using RT-PCR. Unpaired Student's t-tests were performed. **d-e**, Control (sgNTC) or *Cpt1a*-KO (sgCpt1a) PyMT cells were treated with vehicle (VEH) or dideoxycytidine (ddC) to deplete mtDNA. n=3 per group. **d**, Relative levels of mtDNA regions (left) or nDNA (right) in the cytosolic fraction was determined using RT-PCR. Two-way ANOVA with Tukey's post hoc; p=8.48x10^{-4} (Dloop1), p=1.70x10^{-4} (mt-16S), p=0.00129 (mt-Nd4), p=0.757 (Tert). **e**, Relative expression of *Ifi44, Irf7, Lcn2*, and *Slpi* was determined by RT-PCR. Two-way ANOVA with Tukey's post hoc; p=7.57x10^{-4} (Ifi44), p=0.0103 (Irf7), p=0.0319 (Lcn2), p=0.00128 (Slpi). **f-g**, Control (sgNTC) or *Cpt1a*-KO (sgCpt1a) 4T1 cells were transfected with scrambled control (Ctrl), Vdac1, or CypD (Ppif) si RNA. **f**, Immunoblot confirms CypD and VDAC levels. Tubulin serves as loading control. **g**, Relative expression of *Irf7, Ifi44,* and *Lcn2* was determined by RT-PCR. Two-way ANOVA with Tukey's post hoc; p=0.00231 (Irf7), p=6.37x10^{-5} (Ifi44), p=0.00132 (Lcn2 *p<0.05, **p<0.01, ***p<0.005, ****p<0.001. ns, not significant.

Supplementary Table 1. Composition of the metabolism-focused sgRNA library and single gene KO sgRNA used in this study

Gene Symbol	sgRNA ID	sgRNA Sequence (5'→3')
Cpt1a	Cpt1a_sg1	CACATTGTCGTGTACCACAG
Cpt1a	Cpt1a_sg2	CATACTGCTGTATCGTCGCA
Cpt1a	Cpt1a_sg3	ACCTTGGACCCAAATTGCAG
Cpt1a	Cpt1a_sg4	ACGTTGGACGAATCGGAACA
Ppif	Ppif_sg1	GATGTCGTGCCAAAGACTGC
Ppif	Ppif_sg2	CCGCTCGTGTACTTGGACGT
NTC	NTC_sg1	AAAAAGTCCGCGATTACGTC
NTC	NTC_sg2	AAAACGGCTCGATCGGTGAT
NTC	NTC_sg3	AAAACGTAATTATACCGAGC
NTC	NTC_sg4	AAAATTGCACCTTCCCGGCC

Supplementary Table 2. Composition of the metabolism-focused sgRNA library and single gene KO sgRNA used in this study

Gene Symbol	sgRNA ID	sgRNA Sequence (5'→3')
Acaa1b	Acaa1b_sg1	AACCACTGTCCTGAATGACA
Acaa1b	Acaa1b_sg2	GGCGGGAAGAAATATTCCCA
Acaa1b	Acaa1b_sg3	TGTGTGCACTAGGATAACTT
Acaa1b	Acaa1b_sg4	GGAGCCAGAGAGATTACCTC
Acaa2	Acaa2_sg1	GCCCACGATGACACTATCGA
Acaa2	Acaa2_sg2	GCTGAGGTCGTCTTGTGTGG
Acaa2	Acaa2_sg3	CGAGGCTGGCTACTTCAATG
Acaa2	Acaa2_sg4	CGTCCGTGTTCAAGAAAGAC
Acaca	Acaca_sg1	GCCATTCATTATCACTACGT
Acaca	Acaca_sg2	AATGCATGCGATCTATCCGT
Acaca	Acaca_sg3	TTGATTCATAGGTACCGAAG
Acaca	Acaca_sg4	AAGCCCTTCGAACATACACC
Acly	Acly_sg1	GAGAGAGATTGACCCCGACG
Acly	Acly_sg2	AGAGCGATTCGAGATTACCA
Acly	Acly_sg3	TTGTCACCTGTACACGACGG
Acly	Acly_sg4	GGACGAAAAGCTGAATACCG
Aco1	Aco1_sg1	CCACCGCCATTTAGGCCGCG
Aco1	Aco1_sg2	CATATGCTATTACCAGAGGG
Aco1	Aco1_sg3	TAGCCCACCACATCAAACCT
Aco1	Aco1_sg4	GTGTAGCACCTCCGACAAGT
Acss2	Acss2_sg1	GCTGGGAACCTACTACCCGG
Acss2	Acss2_sg2	CAGAACGCCGGTGCAGCTCG
Acss2	Acss2_sg3	AAGGGAAAATATTCACTGAG
Acss2	Acss2_sg4	GCATTGTGGTCAAACATCTG
Adpgk	Adpgk_sg1	CTCTCACGACCTCTCCAACG
Adpgk	Adpgk_sg2	ATGCTGCTTTAATTGGACAG
Adpgk	Adpgk_sg3	CCAGGTACTCTAAAATAAGG
Adpgk	Adpgk_sg4	GCTGGCCAGTATGACCAACA
Ahcy	Ahcy_sg1	GCGCACCTGACAGAAGCTGT
Ahcy	Ahcy_sg2	TGTCAACGATTCTGTCACCA
Ahcy	Ahcy_sg3	TGACCCTATCATACCCTCCA
Ahcy	Ahcy_sg4	TGTGATGATTGCGGGCAAGG
Ahr	Ahr_sg1	AGCTGTGCACAAGAGGATCG
Ahr	Ahr_sg2	GTATAATAGACTGCTGCCTG
Ahr	Ahr_sg3	TCTCCGGTAGCAAACATGAA
Ahr	Ahr_sg4	GTGGAAAGAATCCTTACTTG

Aldob	Aldob_sg1	TATCCACAGTTGGACCAAGG
Aldob	Aldob_sg2	AATTCCATTAGCCAGAGCAT
Aldob	Aldob_sg3	CCGCCTGCAAAGGATAAAGG
Aldob	Aldob_sg4	GGTCCCTATTGTTGAGCCAG
Arg1	Arg1_sg1	AATAAACTTACTGTTCCCCA
Arg1	Arg1_sg2	AGTATGACGTGAGAGACCAC
Arg1	Arg1_sg3	AAATGACACATAGGTCAGGG
Arg1	Arg1_sg4	AGATGTACCAGGATTCTCCT
Arg2	Arg2_sg1	CCAATGTACACAATATTTGG
Arg2	Arg2_sg2	TACACAACCAGATTATTGTA
Arg2	Arg2_sg3	TTTCCAGATACAGTGGTGAG
Arg2	Arg2_sg4	GTCCACTCTGTAGCTATAGT
Ass1	Ass1_sg1	TAACATCTTACCTTAATCTG
Ass1	Ass1_sg2	TGGGATCCTGGAAAACCCCA
Ass1	Ass1_sg3	AAGGCACTTCCTCACCAGGT
Ass1	Ass1_sg4	TGCCTTCACCTGTAGCAACA
Atg16l1	Atg16l1_sg1	CATACTTACGAAGACATACG
Atg16l1	Atg16l1_sg2	CGAACTGCACAAGAAGCGTG
Atg16l1	Atg16l1_sg3	GAAACTGAGGAAAACTACTG
Atg16l1	Atg16l1_sg4	TGCAAGCCGAATCTGGACTG
Atg3	Atg3_sg1	GTAGATACATATCACAACAC
Atg3	Atg3_sg2	GACAGGCTACCCTAGACACA
Atg3	Atg3_sg3	GGGTGTAATCACCCCAGAAG
Atg3	Atg3_sg4	TAACAGTTCCATGCTACAAG
Atg5	Atg5_sg1	AAGAGTCAGCTATTTGACGT
Atg5	Atg5_sg2	AAATGTACTGTGATGTTCCA
Atg5	Atg5_sg3	CCTTCTACACTGTCCATCCA
Atg5	Atg5_sg4	AAGAAAAACTCACCATTTCA
Atg7	Atg7_sg1	TCTCCTACTCCAATCCCGTG
Atg7	Atg7_sg2	TGGGGTCCATACATCCACTG
Atg7	Atg7_sg3	CTTAAAAGCCTCAAGTGTGT
Atg7	Atg7_sg4	CTTGAATAAGAAGTAGGGCA
Atp5a1	Atp5a1_sg1	TGGTCAGAAGCGGTCCACTG
Atp5a1	Atp5a1_sg2	GCTCCCGCACAGAGATTCGG
Atp5a1	Atp5a1_sg3	ACTGGGCGTGTGTTAAGCAT
Atp5a1	Atp5a1_sg4	CCAACAGCTCCTCGCCAACG
Atp5b	Atp5b_sg1	CTGCTGGCCCCATACGCCAA
Atp5b	Atp5b_sg2	GCGCTTACCAGGATGAACCC
Atp5b	Atp5b_sg3	AAATACAGAGTAACCACCAT
Atp5b	Atp5b_sg4	CCCACCCTAGCCACCGACAT

Atp6ap1	Atp6ap1_sg1	GAGGATTTCACAGCATACGG
Atp6ap1	Atp6ap1_sg2	GATATGACCCTCATGTGTGT
Atp6ap1	Atp6ap1_sg3	TAGCTAGATCCACATGCAAG
Atp6ap1	Atp6ap1_sg4	GTGTCATTGTAACTCACAGG
Batf	Batf_sg1	AGAGATCAAACAGCTCACCG
Batf	Batf_sg2	AGGACTCATCTGATGATGTG
Batf	Batf_sg3	GTGGGTACTCACCAGGTGAA
Batf	Batf_sg4	AGGGGGTACCTGTTTGCCAG
Bcl6	Bcl6_sg1	TCTCCACGACCTCACGACCT
Bcl6	Bcl6_sg2	ATGTTGCTGTGACACAAACT
Bcl6	Bcl6_sg3	AATGCACCCTTGAACCGGAA
Bcl6	Bcl6_sg4	GAGGGAAGGCAATATCATGG
Cd5l	Cd5l_sg1	GGCTGATATGATGCGCCACG
Cd5l	Cd5l_sg2	CAACGGAACGGAAGACACGT
Cd5l	Cd5l_sg3	AGCTGCAACAAGAATACTCA
Cd5l	Cd5l_sg4	ACCAAAGTGCAGCTAGTGGG
Cers4	Cers4_sg1	TTGAGAATCTTACAACCCTG
Cers4	Cers4_sg2	CGCTTCGGCAGACTCAACGC
Cers4	Cers4_sg3	GTTACCACCCAATGTCACAT
Cers4	Cers4_sg4	GCGGAGCAAGTTGACGCTGT
Cox10	Cox10_sg1	TCTGTCCCGGAAGCCAAATG
Cox10	Cox10_sg2	GGGAGTGAATCCACTCACAG
Cox10	Cox10_sg3	TATACAGGGATTGCCACACA
Cox10	Cox10_sg4	AGTTGGCAGCACAGGATGCG
Cox15	Cox15_sg1	AGAAAGGGTTGGCTCAACCG
Cox15	Cox15_sg2	AGGCGGTACTGACTGACCCG
Cox15	Cox15_sg3	GGTACATGGAATACTCACAC
Cox15	Cox15_sg4	TAGGGTGGCGTCCAGAACAC
Cox7a1	Cox7a1_sg1	GACCTCCCAGTACACTTGAA
Cox7a1	Cox7a1_sg2	AAGCCACTTAGAAAACCGTG
Cox7a1	Cox7a1_sg3	TGGTAGATGAGCTAAAAGAC
Cox7a1	Cox7a1_sg4	AAAAGACCGGACCAGAGCCT
Cps1	Cps1_sg1	TGAGCCTCACAATTTCGTCG
Cps1	Cps1_sg2	ATGCAGACCGAATCATCACA
Cps1	Cps1_sg3	TACAGTATTCCATGGAAGTG
Cps1	Cps1_sg4	GTTGGTGGCATCTCGTGTCG
Cpt1a	Cpt1a_sg1	CACATTGTCGTGTACCACAG
Cpt1a	Cpt1a_sg2	CATACTGCTGTATCGTCGCA
Cpt1a	Cpt1a_sg3	ACCTTGGACCCAAATTGCAG
Cpt1a	Cpt1a_sg4	ACGTTGGACGAATCGGAACA

Dgat2	Dgat2_sg1	GATCTGCCCTGTCACGCGAG
Dgat2	Dgat2_sg2	CTGGCTCAACAGATCTAAGG
Dgat2	Dgat2_sg3	AAGGCCCTATTTGGCTACGT
Dgat2	Dgat2_sg4	GTCTCGGAAGTAGCGCCACA
Dhfr	Dhfr_sg1	GACATGGTTTGGATAGTCGG
Dhfr	Dhfr_sg2	AACCTCAGAGAACCACCACG
Dhfr	Dhfr_sg3	TCGCCGTGTCCCAAAATATG
Dhfr	Dhfr_sg4	CAGCCCGGCCAATACCTGAG
Fasl	Fasl_sg1	AGGACCACAACACAAATCTG
Fasl	Fasl_sg2	CTTCACTCCAGAGATCAGAG
Fasl	Fasl_sg3	CCTCTGAAAAAAAAGAGCCG
Fasl	Fasl_sg4	GGAACTGGCAGAACTCCGTG
Fasn	Fasn_sg1	CTACCAGGCCATCCGTAGTG
Fasn	Fasn_sg2	TGTCTCCGAAAAGAGCCGGG
Fasn	Fasn_sg3	TTGGTGGAGCCAATTAACAG
Fasn	Fasn_sg4	ACTGGCAATCTGATTGTGAG
Fbp1	Fbp1_sg1	CATGGCAAGGACCAACATGG
Fbp1	Fbp1_sg2	CACAAGAACACAGGTAGCGT
Fbp1	Fbp1_sg3	TGGCTCAACCAATGTGACTG
Fbp1	Fbp1_sg4	AACATCTACAGCCTTAATGA
Foxp3	Foxp3_sg1	CATACCTGATGCATGAAGTG
Foxp3	Foxp3_sg2	TCTACCCACAGGGATCAATG
Foxp3	Foxp3_sg3	AGGTCGGGACCTGCGAAGTG
Foxp3	Foxp3_sg4	GCAAGAGCTCTTGTCCATTG
G6pdx	G6pdx_sg1	AGAGGTGGAAACTGACAACG
G6pdx	G6pdx_sg2	TGCCCGCTCACGACTCACAG
G6pdx	G6pdx_sg3	ATGACCCCACAGTACCCCAT
G6pdx	G6pdx_sg4	AGAGATGGTCCAGAATCTCA
Gapdh	Gapdh_sg1	GCTGTGGCGTGATGGCCGTG
Gapdh	Gapdh_sg2	AAACAGGCCCACTTGAAGGG
Gapdh	Gapdh_sg3	TGCCATTTGCAGTGGCAAAG
Gapdh	Gapdh_sg4	GGCCGGTGCTGAGTATGTCG
Gata3	Gata3_sg1	CTACTACGGAAACTCCGTCA
Gata3	Gata3_sg2	CCGGGTTCGGATGTAAGTCG
Gata3	Gata3_sg3	GCAGCTGCACCTGATACTTG
Gata3	Gata3_sg4	TCCAAGACGTCCATCCACCA
Gclc	Gclc_sg1	TGTGCCGGTCCTTGACTGCG
Gclc	Gclc_sg2	CAATATGAGGAAACGCCGGA
Gclc	Gclc_sg3	AGAAACATCCGGCATCGGAG
Gclc	Gclc_sg4	TGTAGATGATAGAACACGGG

Glo1	Glo1_sg1	CGATCCAGACCCTAGCACCA
Glo1	Glo1_sg2	GCACTGCGTGAGCTCAAGGG
Glo1	Glo1_sg3	GGATAAGAACGATATCCCCA
Glo1	Glo1_sg4	GAGACTCAGAGTTACCACAA
Gls	Gls_sg1	CGACGCGTTCGGCAACAGCG
Gls	Gls_sg2	TGTACATCGCTATGTTGGGA
Gls	Gls_sg3	GATTGCGAACATCTGATCCC
Gls	Gls_sg4	ATATAACTCATCGATGTGTG
Gls2	Gls2_sg1	CGTCCGGTACTACCTCGGTG
Gls2	Gls2_sg2	GGGGATCGGAATTACGCCAT
Gls2	Gls2_sg3	AAAAGCAGGTCACCAAGTCG
Gls2	Gls2_sg4	TGAGTCAGGCAGTGTCATGG
Got1	Got1_sg1	GATCCCCGCAAGGTTAACCT
Got1	Got1_sg2	GTTGGTGATGATACGTAGAT
Got1	Got1_sg3	AGACCTAGAGAAAGATGCGT
Got1	Got1_sg4	CATTCGGCCCTATTGCTACT
Got2	Got2_sg1	TGGAGGTCCCATTTCAACAT
Got2	Got2_sg2	TTTCTGCCCAAACCATCCTG
Got2	Got2_sg3	CATCCTCCTCACCTTCACCA
Got2	Got2_sg4	AGCTCACCTTCCGGACACTG
Gpt2	Gpt2_sg1	GCGGTGGAGTACGCTGTGCG
Gpt2	Gpt2_sg2	ACGCTAAGAAACGAGCGCGG
Gpt2	Gpt2_sg3	GTTCTCTGCATTATCAACCC
Gpt2	Gpt2_sg4	GGGGATGGGAATCATCACGC
Hif1a	Hif1a_sg1	TGAACATCAAGTCAGCAACG
Hif1a	Hif1a_sg2	ATAACGTGAACAAATACATG
Hif1a	Hif1a_sg3	GTGAGAAAACTTCTGGATGC
Hif1a	Hif1a_sg4	AGTAAGAAAATTTCATATCG
Hk1	Hk1_sg1	CCGACAATCCAAAATAGACG
Hk1	Hk1_sg2	CGTAGCCGCCATTGAAACGT
Hk1	Hk1_sg3	GGATCTTTACCAGTAGGACT
Hk1	Hk1_sg4	CTCCCGGGATTATAACCCAA
Hk2	Hk2_sg1	ATTCCCGAGGACATCATGCG
Hk2	Hk2_sg2	GGAGATGCGTCACATTGACA
Hk2	Hk2_sg3	ATCCGGAGTTGACCTCACAA
Hk2	Hk2_sg4	GGAGTGGCACACACATAAGT
Idh1	Idh1_sg1	CCCAGCCTGTCACTAGCCGG
Idh1	Idh1_sg2	GGCTATAAAGAAATACAACG
Idh1	Idh1_sg3	AATTCAAGTTGAAACAAATG
Idh1	Idh1_sg4	TGGTACATGACTTTGAAGGT

Idh2	Idh2_sg1	GGCCACCCAGAAGTACAGTG
Idh2	Idh2_sg2	TCGAGCTGGCACGTTCAAGT
Idh2	Idh2_sg3	TCACCGTCCATCTCCACTAC
Idh2	Idh2_sg4	ACATCGGCTCATCGACGACA
Ikzf2	Ikzf2_sg1	CCTAATTGAGAGCAGCGAGG
Ikzf2	Ikzf2_sg2	GCTTGTCATGTGACTTGGCG
Ikzf2	Ikzf2_sg3	TATGAACTTAACATATGAGA
Ikzf2	Ikzf2_sg4	GGGTAAAAGAAGCTCCGCAC
Il2ra	Il2ra_sg1	GTGTCTGTATGACCCACCCG
Il2ra	Il2ra_sg2	ATCTTGCAGATGCTAATAGC
Il2ra	Il2ra_sg3	GAGAGGTTTCCGAAGACTAA
Il2ra	Il2ra_sg4	GAATCTTCATGTTTCCAAGG
Kdsr	Kdsr_sg1	ACCATGAAGGAGCGACGGGT
Kdsr	Kdsr_sg2	ACAGTGACGTACACATTGTA
Kdsr	Kdsr_sg3	AGTGGAGAATGTCATAAAGC
Kdsr	Kdsr_sg4	GCTATTGAGTGCTACAAACA
Ldha	Ldha_sg1	CAAGCTGGTCATTATCACCG
Ldha	Ldha_sg2	GTTGCAATCTGGATTCAGCG
Ldha	Ldha_sg3	GGAGAACATGGCGACTCCAG
Ldha	Ldha_sg4	GTCATGGAAGACAAACTCAA
Mdh1	Mdh1_sg1	GTCAGCGCCATCGATCCCCA
Mdh1	Mdh1_sg2	GTCCATAGATGTCATTGCAA
Mdh1	Mdh1_sg3	GACATTCTTTACATCATCAG
Mdh1	Mdh1_sg4	GTCTTTGGGAAAGACCAGGT
Mthfd1	Mthfd1_sg1	ACACCAACGATAGATTCCTG
Mthfd1	Mthfd1_sg2	CACTATGAATCCGTGCACAG
Mthfd1	Mthfd1_sg3	GATTGCCGGAAGGCACGCGG
Mthfd1	Mthfd1_sg4	GGTAGCGTCCAGTAAGAAAG
Mthfd2	Mthfd2_sg1	TCGATGAGATATTGTGACTG
Mthfd2	Mthfd2_sg2	AGATAATTAAGCGAACAGGT
Mthfd2	Mthfd2_sg3	GCTTTCATGTCATTAACGTG
Mthfd2	Mthfd2_sg4	CTATGTTCTCAACAAAACCA
Ndufc1	Ndufc1_sg1	TTGGCAGTTGGACTGTCCGT
Ndufc1	Ndufc1_sg2	CGAAAACGAGCGCAGCACTA
Ndufc1	Ndufc1_sg3	CACGGTCGAAGTTCTATGTC
Ndufc1	Ndufc1_sg4	CAATGCCAAACCTAACTGGT
Nfe2l2	Nfe2l2_sg1	TGAAGACTGAACTTTCAGCG
Nfe2l2	Nfe2l2_sg2	GTTCTGTTTGACACTTCCAG
Nfe2l2	Nfe2l2_sg3	TTCAACCCGAAGCACGCTGA
Nfe2l2	Nfe2l2_sg4	GGTGGGATTTGAGTCTAAGG

Olah	Olah_sg1	AAAACCAGAACTTACGTGAG
Olah	Olah_sg2	TGCATGCTGTAAGACTGGCT
Olah	Olah_sg3	TTACAAGATCTAAATACCTG
Olah	Olah_sg4	ATTAATCTTTCGGCCCCACT
Otc	Otc_sg1	CAGTCCATTGACAATTGGGA
Otc	Otc_sg2	CCTTCAAGCAGCTACTCCAA
Otc	Otc_sg3	TAGAAAGGGTCACACTTCTG
Otc	Otc_sg4	AAATTCAGGATCAAGCAGAA
Pck1	Pck1_sg1	ACTGACAGACTCGCCCTATG
Pck1	Pck1_sg2	GTGGCCGAGACTAGCGATGG
Pck1	Pck1_sg3	CCTTTGGAAGCGGATATGGT
Pck1	Pck1_sg4	TCGCAGATGTGGATATACTC
Pck2	Pck2_sg1	TGCGTATTATGACCCGCCTG
Pck2	Pck2_sg2	TGATTGTAACTCCTTCGCAG
Pck2	Pck2_sg3	AGGGTTTGGATGCTACGGCA
Pck2	Pck2_sg4	ATGGAAGCACATACATAATG
Pdcd1	Pdcd1_sg1	CAATACAGGGATACCCACTA
Pdcd1	Pdcd1_sg2	GACACACGGCGCAATGACAG
Pdcd1	Pdcd1_sg3	CAGCTTGTCCAACTGGTCGG
Pdcd1	Pdcd1_sg4	GCTCAAACCATTACAGAAGG
Pdk1	Pdk1_sg1	TTGTCGCAGAAACATAAACG
Pdk1	Pdk1_sg2	ATGGCTATGAGAACGCTAGG
Pdk1	Pdk1_sg3	TTGATAGCCTTATTGTTCGG
Pdk1	Pdk1_sg4	AAACACCATGTGATAGAGAT
Pfkm	Pfkm_sg1	CCTCACGGTAGAGCGAACAG
Pfkm	Pfkm_sg2	GCGCCTTGGATATGACACCC
Pfkm	Pfkm_sg3	TTAGACCAAAGACGTGACCA
Pfkm	Pfkm_sg4	CATAGACACGCTCTCCCACG
Pgk1	Pgk1_sg1	TAAGGTGCTCAACAACATGG
Pgk1	Pgk1_sg2	TCAAGAACAGAACATCCCTG
Pgk1	Pgk1_sg3	GGACTGCACACCGAGCCCAT
Pgk1	Pgk1_sg4	CTTCCTCTACATGAAAGCGG
Pgk2	Pgk2_sg1	GGATACCATCAGGCCGACCG
Pgk2	Pgk2_sg2	GGATGATAGACCCATTATCT
Pgk2	Pgk2_sg3	CGGGCTCACAGTTCTACGGT
Pgk2	Pgk2_sg4	GGAAGGCTTCTACTTTAGCA
Pgm1	Pgm1_sg1	CATTACCGATGGACGCGCTG
Pgm1	Pgm1_sg2	ATCATCTCTCCCCACGATCG
Pgm1	Pgm1_sg3	TGGGGGTTATATCAGAGAAG
Pgm1	Pgm1_sg4	AGGCCAACTGCACAAACTCG

Pgm2	Pgm2_sg1	CGGCCGCTTCTACATGACCG
Pgm2	Pgm2_sg2	CGCATAGACGCCATGCACGG
Pgm2	Pgm2_sg3	CAGCCAGCCATAATCCAGGA
Pgm2	Pgm2_sg4	CAGCAGCATAGGTGAGATTG
Pik3c3	Pik3c3_sg1	AGCCTGTAAGAACTCAACAC
Pik3c3	Pik3c3_sg2	ATACACATCCCATATAGTCA
Pik3c3	Pik3c3_sg3	CTCACCAAGGCTCATCGGCA
Pik3c3	Pik3c3_sg4	ATGGACCAGGCGATCTACAA
Pkm	Pkm_sg1	TTTCTCTCATGGAACCCATG
Pkm	Pkm_sg2	TGAAATAGCACATGCCTGTG
Pkm	Pkm_sg3	GGGCAGAGTCAATGTCCAGG
Pkm	Pkm_sg4	CTTCCTGACTTCATGCACGT
Ppat	Ppat_sg1	ACCTTGGAATCGGACATACG
Ppat	Ppat_sg2	ATAAGACGCCCGATGCAGAG
Ppat	Ppat_sg3	TGATCACTCTGGGACTCGTG
Ppat	Ppat_sg4	AGGGGTGTATGCGAGTAACT
Prdx2	Prdx2_sg1	ATCAAGCTTTCGGACTACAG
Prdx2	Prdx2_sg2	CCTTCAGGATCAATACCCCA
Prdx2	Prdx2_sg3	GCTAAAAGCGATGATCTCCG
Prdx2	Prdx2_sg4	CTTCCGAAAGCTAGGCTGCG
Rheb	Rheb_sg1	AACAAACTGAATTGTCAATG
Rheb	Rheb_sg2	CCATATCCAACAACTTGCCA
Rheb	Rheb_sg3	TTCAGCTTGTAGACACAGCG
Rheb	Rheb_sg4	TCATAGGATACCTATTATGT
Rorc	Rorc_sg1	CTTGAGTATAGTCCAGAACG
Rorc	Rorc_sg2	GTCATCTGGGATCCACTACG
Rorc	Rorc_sg3	TCTGGGGCACTGCAGAAACT
Rorc	Rorc_sg4	GACAAGCAGAGGCCTCGGGT
Sdha	Sdha_sg1	GTCAGTTACCTCAACCACAG
Sdha	Sdha_sg2	TTCTACTCAATACCCAGTGG
Sdha	Sdha_sg3	TGCACAGTGCAATGACACCA
Sdha	Sdha_sg4	ACTGTGCATTACAACATGGG
Sdhb	Sdhb_sg1	TGCGCCATGAACATCAACGG
Sdhb	Sdhb_sg2	ACAGTATCTGCAGTCCATCG
Sdhb	Sdhb_sg3	ACCTCGAATGCAGACGTACG
Sdhb	Sdhb_sg4	TAGAAGTTACTCAAATCCTG
Sgpp1	Sgpp1_sg1	TGCCTAAGTAGAATCTACAT
Sgpp1	Sgpp1_sg2	TGAGCAGGAACATGGCGATG
Sgpp1	Sgpp1_sg3	CCTCGCCCGTCAACGAGTTG
Sgpp1	Sgpp1_sg4	TGGGTGCTGGTCATGTACCT

Shmt1	Shmt1_sg1	TGTAGAATATCATACCAGCA
Shmt1	Shmt1_sg2	TCGGCTGGCAAAATTCTCCG
Shmt1	Shmt1_sg3	CCCGGAACCTGGACTACGCA
Shmt1	Shmt1_sg4	AAGCCCATGATTCGCCCATG
Shmt2	Shmt2_sg1	CGGCAGATACTACGGAGGAG
Shmt2	Shmt2_sg2	AACATCCGCGTACTTGAAAG
Shmt2	Shmt2_sg3	AGCCTCATGATCGAATCATG
Shmt2	Shmt2_sg4	TAGTCGATGAGGCCAGTTTG
Slc16a1	Slc16a1_sg1	ACTACTAAGAAAGACCAAAG
Slc16a1	Slc16a1_sg2	CACCAGCGATCATTACTGGA
Slc16a1	Slc16a1_sg3	GACTTGCAGCCAACACCAAG
Slc16a1	Slc16a1_sg4	AGGCCCTATTGGTCTCATCA
Slc16a7	Slc16a7_sg1	ATTACCTCCAATGAAGCCAA
Slc16a7	Slc16a7_sg2	AGAGGTACTGGATTCGTGGA
Slc16a7	Slc16a7_sg3	GCTCAGTACGCTAAACACAT
Slc16a7	Slc16a7_sg4	TTCACCAACACACTACTGAT
Slc1a5	Slc1a5_sg1	AATCCCTATCGATTCCTGTG
Slc1a5	Slc1a5_sg2	TACAACAGAGTCGTTGATGG
Slc1a5	Slc1a5_sg3	GCGGGAGATCAATTCAACCA
Slc1a5	Slc1a5_sg4	GTGGTGTGCAGCCTGATCGG
Slc20a1	Slc20a1_sg1	GTAGAAAGGTTACCTTACGG
Slc20a1	Slc20a1_sg2	TCAGTATCACACCGTGCACA
Slc20a1	Slc20a1_sg3	CCGGAACGGCTTGATAGATG
Slc20a1	Slc20a1_sg4	GCCACATATTGCCATAGTGT
Slc2a1	Slc2a1_sg1	CCTGCTCATCAATCGTAACG
Slc2a1	Slc2a1_sg2	TCAGCATGGAGTTCCGCCTG
Slc2a1	Slc2a1_sg3	GTGTCACCTACAGCTCTACG
Slc2a1	Slc2a1_sg4	CAAACATGGAACCACCGCTA
Slc38a1	Slc38a1_sg1	ATACTTTGGTGTGCACGCGT
Slc38a1	Slc38a1_sg2	TGCATGGTGTATGAGAAGCT
Slc38a1	Slc38a1_sg3	TCACCATCACCACCAACACT
Slc38a1	Slc38a1_sg4	AGATTGGCAGGACGGACGGG
Slc38a2	Slc38a2_sg1	CCACCAAAGCAGCTTCCACG
Slc38a2	Slc38a2_sg2	CTCAAGACTGCCAACGAAGG
Slc38a2	Slc38a2_sg3	GCAGTGACAATGGAAGAATG
Slc38a2	Slc38a2_sg4	GAGTTGAAGATGAAATAGCG
Slc3a2	Slc3a2_sg1	GTTCACCGGCTTATCCAAGG
Slc3a2	Slc3a2_sg2	CGCCCGAACGATGATAACCA
Slc3a2	Slc3a2_sg3	TATCACCAAGAACTTAAGTG
Slc3a2	Slc3a2_sg4	GTACTGAATCCCTAGTCACT

Slc6a1	Slc6a1_sg1	CACCAACATGACCAGCGCCG
Slc6a1	Slc6a1_sg2	GCAGAAATACACGAGCACCC
Slc6a1	Slc6a1_sg3	TACCTCTGTGGGAAAAACGG
Slc6a1	Slc6a1_sg4	TCCATGTGTCCCGGTCAGGG
Slc7a1	Slc7a1_sg1	GCCATGGCATAGATAACTCG
Slc7a1	Slc7a1_sg2	CACAAACGTGAAATACGGTG
Slc7a1	Slc7a1_sg3	TGACGTGAGAACTCTCCGAT
Slc7a1	Slc7a1_sg4	CCAGGTCCTTCAGTTCAAAG
Smox	Smox_sg1	CGAGAGTCAGAACAGCGTCG
Smox	Smox_sg2	CAACTCGCATGAAGCCCGAG
Smox	Smox_sg3	GCTCGATCTCAGGACCCCGG
Smox	Smox_sg4	GCCTGCTACCTTACCAACCG
Sod1	Sod1_sg1	CAGTATGGGGACAATACACA
Sod1	Sod1_sg2	GACTGCTGGAAAGGACGGTG
Sod1	Sod1_sg3	TAAGAAACATGGTGGCCCGG
Sod1	Sod1_sg4	AAAGCGGTGTGCGTGCTGAA
Sod2	Sod2_sg1	GGCGTTGAGATTGTTCACGT
Sod2	Sod2_sg2	ATGATCTGCGCGTTAATGTG
Sod2	Sod2_sg3	ACAAACCTGAGCCCTAAGGG
Sod2	Sod2_sg4	CCTGCACTGAAGTTCAATGG
Sptlc1	Sptlc1_sg1	AATGTGCCATAGAACCCTCG
Sptlc1	Sptlc1_sg2	CCCTCCAACCCACAACATCG
Sptlc1	Sptlc1_sg3	TCCTGCGTACTCTAAGAGAG
Sptlc1	Sptlc1_sg4	TTTGTCGTAGAATCCTCGCA
Sptlc2	Sptlc2_sg1	GTTGTGTTTGAAGATTCGAA
Sptlc2	Sptlc2_sg2	TGAGAGCAATCACTTCAGGA
Sptlc2	Sptlc2_sg3	AATCTCGAAGATATCCAAAG
Sptlc2	Sptlc2_sg4	ACAACTATCTTGGATTTGCG
Sptssa	Sptssa_sg1	CAGGTACTGGTAGTAGAACC
Sptssa	Sptssa_sg2	CTGAACACGGTTCGCTCCCA
Sptssa	Sptssa_sg3	CCATCACGCAGATTCGATGC
Sptssa	Sptssa_sg4	GTACAGGGCCATCCCCACCA
Tbx21	Tbx21_sg1	AGTCTGGGTGGACATATAAG
Tbx21	Tbx21_sg2	AGGACTACGCATTGCCCGCG
Tbx21	Tbx21_sg3	GACCCGACCGATCGCCGCGC
Tbx21	Tbx21_sg4	GGCTTCCAACAATGTGACCC
Uqcrh	Uqcrh_sg1	TGGATCTGGAGACCCCAAAG
Uqcrh	Uqcrh_sg2	GACGAACGAAAGATGCTCAC
Uqcrh	Uqcrh_sg3	AATCCTCTTCTGTCTGTGAC
Uqcrh	Uqcrh_sg4	GCTCTCTCACTGTTGTTAGG

Tsc2	Tsc2_sg1	TGAACCACATGGCTATGACG
Tsc2	Tsc2_sg2	CACAGGGTGATAATGAACAG
Tsc2	Tsc2_sg3	CAGCTCCAAAGACCCTTGAG
Tsc2	Tsc2_sg4	CTGATCCTAGCACACATGTG
Aldoa	Aldoa_sg1	AATGGCGAGACAACTACCCA
Aldoa	Aldoa_sg2	CCTTGCCCGGAGCCACAATG
Aldoa	Aldoa_sg3	CCACGAGACACTGTACCAGA
Aldoa	Aldoa_sg4	GCCAGCATCTGCCAGCAGGT
NTC	NTC_sg1	AAAAAGTCCGCGATTACGTC
NTC	NTC_sg2	AAAACGGCTCGATCGGTGAT
NTC	NTC_sg3	AAAACGTAATTATACCGAGC
NTC	NTC_sg4	AAAATTGCACCTTCCCGGCC
NTC	NTC_sg5	AAACCCCCGCGCGGAGCGTC
NTC	NTC_sg6	AAACCTAGCGTAGATTCGGC
NTC	NTC_sg7	AAACGAGGCTGTTCGTACAC
NTC	NTC_sg8	AAACTCATACGTAGCGAATC

Supplementary Table 3. NGS PCR2 indexing primers for pooled CRISPR screen sequencing

Primer Name	Full Primer Sequence (5'→3')	Adapter Type	Index Barcode Sequence	Notes
LenNGS-Fwd-1	AATGATACGGCGACCACCGAGATCTACACTCTTTCCCTACACGACGCTCTTCCGATCTTAAGTAGAGGCTTTATATATCTTGTGGAAAGGACGAAACACC	P5	TAAGTAGAG	Forward indexing primer
LenNGS-Fwd-2	AATGATACGGCGACCACCGAGATCTACACTCTTTCCCTACACGACGCTCTTCCGATCTATCATGCTTAGCTTTATATATCTTGTGGAAAGGACGAAACACC	P5	ATCATGCTTA	Forward indexing primer
LenNGS-Fwd-3	AATGATACGGCGACCACCGAGATCTACACTCTTTCCCTACACGACGCTCTTCCGATCTGATGCACATCTGCTTTATATATCTTGTGGAAAGGACGAAACACC	P5	GATGCACATCT	Forward indexing primer
LenNGS-Fwd-4	AATGATACGGCGACCACCGAGATCTACACTCTTTCCCTACACGACGCTCTTCCGATCTCGATTGCTCGACGCTTTATATATCTTGTGGAAAGGACGAAACACC	P5	CGATTGCTCGAC	Forward indexing primer
LenNGS-Fwd-5	AATGATACGGCGACCACCGAGATCTACACTCTTTCCCTACACGACGCTCTTCCGATCTTCGATAGCAATTCGCTTTATATATCTTGTGGAAAGGACGAAACACC	P5	TCGATAGCAATTC	Forward indexing primer
LenNGS-Fwd-6	AATGATACGGCGACCACCGAGATCTACACTCTTTCCCTACACGACGCTCTTCCGATCTATCGATAGTTGCTTGCTTTATATATCTTGTGGAAAGGACGAAACACC	P5	ATCGATAGTTGCTT	Forward indexing primer
LenNGS-Fwd-7	AATGATACGGCGACCACCGAGATCTACACTCTTTCCCTACACGACGCTCTTCCGATCTGATCGATCCAGTTAGGCTTTATATATCTTGTGGAAAGGACGAAACACC	P5	GATCGATCCAGTTAG	Forward indexing primer
LenNGS-Fwd-8	AATGATACGGCGACCACCGAGATCTACACTCTTTCCCTACACGACGCTCTTCCGATCTCGATCGATTTGAGCCTGCTTTATATATCTTGTGGAAAGGACGAAACACC	P5	CGATCGATTTGAGCCT	Forward indexing primer
LenNGS-Fwd-9	AATGATACGGCGACCACCGAGATCTACACTCTTTCCCTACACGACGCTCTTCCGATCTACGATCGATACACGATCGCTTTATATATCTTGTGGAAAGGACGAAACACC	P5	ACGATCGATACACGATC	Forward indexing primer
LenNGS-Fwd-10	AATGATACGGCGACCACCGAGATCTACACTCTTTCCCTACACGACGCTCTTCCGATCTTACGATCGATGGTCCAGAGCTTTATATATCTTGTGGAAAGGACGAAACACC	P5	TACGATCGATGGTCCAGA	Forward indexing primer
LenNGS-Rev-1	CAAGCAGAAGACGGCATACGAGATAAGTAGAGGTGACTGGAGTTCAGACGTGTGCTCTTCCGATCTCCGACTCGGTGCCACTTTTTCAA	P7	AAGTAGAG	Reverse indexing primer
LenNGS-Rev-2	CAAGCAGAAGACGGCATACGAGATACACGATCGTGACTGGAGTTCAGACGTGTGCTCTTCCGATCTCCGACTCGGTGCCACTTTTTCAA	P7	ACACGATC	Reverse indexing primer
LenNGS-Rev-3	CAAGCAGAAGACGGCATACGAGATCGCGCGGTGTGACTGGAGTTCAGACGTGTGCTCTTCCGATCTCCGACTCGGTGCCACTTTTTCAA	P7	CGCGCGGT	Reverse indexing primer
LenNGS-Rev-4	CAAGCAGAAGACGGCATACGAGATCATGATCGGTGACTGGAGTTCAGACGTGTGCTCTTCCGATCTCCGACTCGGTGCCACTTTTTCAA	P7	CATGATCG	Reverse indexing primer
LenNGS-Rev-5	CAAGCAGAAGACGGCATACGAGATCGTTACCAGTGACTGGAGTTCAGACGTGTGCTCTTCCGATCTCCGACTCGGTGCCACTTTTTCAA	P7	CGTTACCA	Reverse indexing primer
LenNGS-Rev-6	CAAGCAGAAGACGGCATACGAGATTCCTTGGTGTGACTGGAGTTCAGACGTGTGCTCTTCCGATCTCCGACTCGGTGCCACTTTTTCAA	P7	TCCTTGGT	Reverse indexing primer
LenNGS-Rev-7	CAAGCAGAAGACGGCATACGAGATAACGCATTGTGACTGGAGTTCAGACGTGTGCTCTTCCGATCTCCGACTCGGTGCCACTTTTTCAA	P7	AACGCATT	Reverse indexing primer
LenNGS-Rev-8	CAAGCAGAAGACGGCATACGAGATACAGGTATGTGACTGGAGTTCAGACGTGTGCTCTTCCGATCTCCGACTCGGTGCCACTTTTTCAA	P7	ACAGGTAT	Reverse indexing primer
LenNGS-Rev-9	CAAGCAGAAGACGGCATACGAGATAGGTAAGGGTGACTGGAGTTCAGACGTGTGCTCTTCCGATCTCCGACTCGGTGCCACTTTTTCAA	P7	AGGTAAGG	Reverse indexing primer
LenNGS-Rev-10	CAAGCAGAAGACGGCATACGAGATAACAATGGGTGACTGGAGTTCAGACGTGTGCTCTTCCGATCTCCGACTCGGTGCCACTTTTTCAA	P7	AACAATGG	Reverse indexing primer
LenNGS-Rev-11	CAAGCAGAAGACGGCATACGAGATACTGTATCGTGACTGGAGTTCAGACGTGTGCTCTTCCGATCTCCGACTCGGTGCCACTTTTTCAA	P7	ACTGTATC	Reverse indexing primer
LenNGS-Rev-12	CAAGCAGAAGACGGCATACGAGATAGGTCGCAGTGACTGGAGTTCAGACGTGTGCTCTTCCGATCTCCGACTCGGTGCCACTTTTTCAA	P7	AGGTCGCA	Reverse indexing primer
LenNGS-Rev-13	CAAGCAGAAGACGGCATACGAGATTCTCATGAGTGACTGGAGTTCAGACGTGTGCTCTTCCGATCTCCGACTCGGTGCCACTTTTTCAA	P7	TCTCATGA	Reverse indexing primer
LenNGS-Rev-14	CAAGCAGAAGACGGCATACGAGATCTCTGCAGGTGACTGGAGTTCAGACGTGTGCTCTTCCGATCTCCGACTCGGTGCCACTTTTTCAA	P7	CTCTGCAG	Reverse indexing primer
LenNGS-Rev-15	CAAGCAGAAGACGGCATACGAGATCACTATCAGTGACTGGAGTTCAGACGTGTGCTCTTCCGATCTCCGACTCGGTGCCACTTTTTCAA	P7	CACTATCA	Reverse indexing primer
LenNGS-Rev-16	CAAGCAGAAGACGGCATACGAGATTGTCGCTGGTGACTGGAGTTCAGACGTGTGCTCTTCCGATCTCCGACTCGGTGCCACTTTTTCAA	P7	TGTCGCTG	Reverse indexing primer
LenNGS-Rev-17	CAAGCAGAAGACGGCATACGAGATGCAGAGCTGTGACTGGAGTTCAGACGTGTGCTCTTCCGATCTCCGACTCGGTGCCACTTTTTCAA	P7	GCAGAGCT	Reverse indexing primer
LenNGS-Rev-18	CAAGCAGAAGACGGCATACGAGATATGAGATCGTGACTGGAGTTCAGACGTGTGCTCTTCCGATCTCCGACTCGGTGCCACTTTTTCAA	P7	ATGAGATC	Reverse indexing primer
LenNGS-Rev-19	CAAGCAGAAGACGGCATACGAGATTTGCTGCCGTGACTGGAGTTCAGACGTGTGCTCTTCCGATCTCCGACTCGGTGCCACTTTTTCAA	P7	TTGCTGCC	Reverse indexing primer
LenNGS-Rev-20	CAAGCAGAAGACGGCATACGAGATCCATCATTGTGACTGGAGTTCAGACGTGTGCTCTTCCGATCTCCGACTCGGTGCCACTTTTTCAA	P7	CCATCATT	Reverse indexing primer

Supplementary Table 4. RT-PCR primers used in study.

Gene	Primer Name	Direction	Sequence (5'→3')
Cpt1a	Mus_Cpt1a_Fwd	Forward	AGTGGCCTCACAGACTCCAG
Cpt1a	Mus_Cpt1a_Rev	Reverse	GCCCATGTTGTACAGCTTCC
Rpl37	Mus_Rpl37_Fwd	Forward	CTACCGCAGATTCAGACATGGA
Rpl37	Mus_Rpl37_Rev	Reverse	ACCGAACTCTGAACCGATGT
Gapdh	Mus_Gapdh_Fwd	Forward	TCAGGAGAGTGTTTCCTCGTC
Gapdh	Mus_Gapdh_Rev	Reverse	TTTGCCGTGAGTGGAGTCAT
Lcn2	Mus_Lcn2_Fwd	Forward	ACTCTGGGAAATATGCACAGGTAT
Lcn2	Mus_Lcn2_Rev	Reverse	AAGCGGGTGAAACGTTCCTT
Slpi	Mus_Slpi_Fwd	Forward	TTGAGAAGCCACAATGCCGT
Slpi	Mus_Slpi_Rev	Reverse	GAGTTTTGACGCACCTCCCA
Cxcl5	Mus_Cxcl5_Fwd	Forward	CCCTACGGTGGAAGTCATAGC
Cxcl5	Mus_Cxcl5_Rev	Reverse	TTAGCTTTCTTTTTGTCACTGCCC
Rsad2	Mus_Rsad2_Fwd	Forward	GTGCCTGAATCTAACCAGAAGATGA
Rsad2	Mus_Rsad2_Rev	Reverse	ATACTTTCCGCCACGCTTCA
Cmpk2	Mus_Cmpk2_Fwd	Forward	GGTAAGACCACACTGACGCA
Cmpk2	Mus_Cmpk2_Rev	Reverse	AGCCACGAGATAATTGCCCA
Ifi44	Mus_Ifi44_Fwd	Forward	GGTACAGACTCTTCACACAGACTT
Ifi44	Mus_Ifi44_Rev	Reverse	TTCTGCACACTCGCCTTGTA
Irf7	Mus_Irf7_Fwd	Forward	CAAGAGAAAATGCTGGGCTCC
Irf7	Mus_Irf7_Rev	Reverse	ATAGGGTTCCTCGTAAACACGG
Tmem173	Mus_Tmem173_Fwd	Forward	GCTGCTGATGCCATACTCCAA
Tmem173	Mus_Tmem173_Rev	Reverse	AGTAGTCCAAGTTCGTGCGAG
Ppif	Mus_Ppif_Fwd	Forward	GATGTCGTGCCAAAGACTGC
Ppif	Mus_Ppif_Rev	Reverse	AGTGTGAAGTTCTCGTCGGG
mtD-loop1	Mus_mtD-loop1_Fwd	Forward	AATCTACCATCCTCCGTGAAACC
mtD-loop1	Mus_mtD-loop1_Rev	Reverse	TCAGTTTAGCTACCCCCAAGTTTAA
mtD-loop2	Mus_mtD-loop2_Fwd	Forward	CCCTTCCCCATTTGGTCT
mtD-loop2	Mus_mtD-loop2_Rev	Reverse	TGGTTTCACGGAGGATGG
mtD-loop3	Mus_mtD-loop3_Fwd	Forward	TCCTCCGTGAAACCAACAA
mtD-loop3	Mus_mtD-loop3_Rev	Reverse	AGCGAGAAGAGGGGCATT
mt16S	Mus_mt16S_Fwd	Forward	CACTGCCTGCCCAGTGA
mt16S	Mus_mt16S_Rev	Reverse	ATACCGCGGCCGTTAAA
mtNd4	Mus_mtNd4_Fwd	Forward	AACGGATCCACAGCCGTA
mtNd4	Mus_mtNd4_Rev	Reverse	AGTCCTCGGGCCATGATT

Supplementary Table 5. Flow cytometry antibodies used in study.

Antibody	Clone	Dilution	Company	Catalog #
APC/Cy7-CD45	30-F11	1:500	BD Biosciences	557659
V450-CD45.2	104	1:500	Tonbo/Cytek	75-0454
PE/Cy7-TCRb	H57-597	1:200	Tonbo/Cytek	60-5961
PerCP/Cy5.5-TCRb	H57-597	1:250	Tonbo/Cytek	65-5961
RedFluor710-CD8	53-6.7	1:500	Tonbo/Cytek	80-0081
BV605-CD8	53-6.7	1:400	Biolegend	100743
PE/Dazzle594-CD4	GK1.5	1:500	Biolegend	100455
PerCP/Cy5.5-CD11b	M1/70	1:500	Tonbo/Cytek	65-0112
APC/Cy7-CD25	PC61	1:200	Biolegend	102025
PerCP/Cy5.5-CD127	A7R34	1:100	eBioscience	45-1271
V450-IFNg	XMG1.2	1:100	Tonbo/Cytek	75-7311
APC-TNFa	MP6-XT22	1:50	eBioscience	17-7321-82
PE-GATA3	TWAJ	1:100	eBioscience	Dec-66
eFluor660-FoxP3	FJK-16s	1:100	eBioscience	50-5773-82
APC-IgG1,k	eBRG1	1:50	eBioscience	17-4301-82
